# Autophagy in Trypanosomatids

**DOI:** 10.3390/cells1030346

**Published:** 2012-07-27

**Authors:** Ana Brennand, Eva Rico, Paul A. M. Michels

**Affiliations:** 1 Research Unit for Tropical Diseases, de Duve Institute, Université catholique de Louvain, Avenue Hippocrate 74, postal box B1.74.01, B-1200 Brussels, Belgium; Email: ana.paesbarreto@uclouvain.be; 2 Department of Biochemistry and Molecular Biology, University Campus, University of Alcalá, Alcalá de Henares, Madrid, 28871, Spain; Email: eva.rico@uah.es

**Keywords:** autophagy, Trypanosomatidae, *Trypanosoma*, *Leishmania*, parasites, life-cycle differentiation, glycosomes, pexophagy, drug discovery, drug action

## Abstract

Autophagy is a ubiquitous eukaryotic process that also occurs in trypanosomatid parasites, protist organisms belonging to the supergroup Excavata, distinct from the supergroup Opistokontha that includes mammals and fungi. Half of the known yeast and mammalian AuTophaGy (ATG) proteins were detected in trypanosomatids, although with low sequence conservation. Trypanosomatids such as *Trypanosoma brucei*, *Trypanosoma cruzi* and *Leishmania* spp. are responsible for serious tropical diseases in humans. The parasites are transmitted by insects and, consequently, have a complicated life cycle during which they undergo dramatic morphological and metabolic transformations to adapt to the different environments. Autophagy plays a major role during these transformations. Since inhibition of autophagy affects the transformation, survival and/or virulence of the parasites, the ATGs offer promise for development of drugs against tropical diseases. Furthermore, various trypanocidal drugs have been shown to trigger autophagy-like processes in the parasites. It is inferred that autophagy is used by the parasites in an—not always successful—attempt to cope with the stress caused by the toxic compounds.

## 1. Introduction

### 1.1. Trypanosomatidae

Trypanosomatidae is a family of protists that belong to the major clade, Kinetoplastea [1]. These are organisms that diverged early from the main eukaryotic branch of the evolutionary tree and are grouped together because of the presence of a single large mitochondrion that extends through most of the body of these unicellular organisms, and whose DNA creates a unique elaborate structure named kinetoplast, located close to the flagellar basal body [2]. Different trypanosomatids have evolved to extremely successful parasites. They can be found over almost the entire planet, being able to parasitize all groups of vertebrates, several species of invertebrates and even plants [3,4,5,6,7]. Among their other peculiarities, Kinetoplastea are characterized by the presence of peroxisome-like organelles named glycosomes, in which the major part of the glycolytic pathway is sequestered, as well as other important metabolic processes and enzymes [8,9]. This unique compartmentalization of glycolysis has been shown to be essential for the survival of the parasites when growing on glucose as carbon source, an obligatory situation for parasites living in sugar-rich environments such as the mammalian bloodstream [8,10,11].

The Trypanosomatidae family includes 13 genera [1,12], all parasitic, among which *Trypanosoma* and *Leishmania* stand out, responsible for diverse clinical manifestations including major human diseases, such as Chagas disease, sleeping sickness and leishmaniasis. Chagas disease consists of a chronic and systemic infection caused by *Trypanosoma cruzi*. The disease affects about eight million people in Latin America, of whom 30%–40% either have or will develop cardiomyopathy, digestive megasyndromes, or both. Nowadays it is also becoming an emerging health problem in non-endemic areas because of increasing population movements [13]. Sleeping sickness, also known as human African trypanosomiasis (or HAT), is caused by *Trypanosoma brucei* and manifests itself as two types of disease: East-African human trypanosomiasis caused by *Trypanosoma brucei rhodesiense* and the West-African form caused by *Trypanosoma brucei gambiense*. Although improved diagnosis and control during the last decade have brought the incidence down to less than 10,000 new cases per year [14], it continues to pose a major threat to 60 million people in 36 countries in sub-Saharan Africa when control measures collapse under political unrest and where drug resistance arises. The disease has an early or hemolymphatic stage, followed by a late or encephalitic stage, when the parasites cross the blood-brain barrier and invade the central nervous system [15]. Finally, leishmaniasis, caused by different parasites belonging to the genus *Leishmania*, is endemic in 88 countries. It is estimated that about 12 million people are currently infected and 350 million more are at risk of contracting the disease [16,17]. Dependent on the *Leishmania* species the disease leads to cutaneous lesions (starting as papules that can eventually ulcerate), mucocutaneous, disfiguring lesions (affecting the nose, oral cavity and pharynx that can cause difficulty in eating and an increased risk for secondary infection and death) [17,18], or fatal, generalized visceral infection (generally causing fever, weight loss, hepatosplenomegaly, lymphadenopathy, pancytopenia and hypergammaglobulinaemia) [19]. Problems related to toxicity, availability, difficulties to administer, high costs and/or emergence of resistance for the currently used drugs make it difficult to provide adequate therapy for the different illnesses caused by trypanosomatids [20,21]. It is therefore necessary to develop new, efficacious and affordable drugs, and consequently to identify new potential targets in the parasites. These targets should be essential for the viability and/or virulence and possess unique features that can be exploited for selective inhibitors that will not affect the human host cells.

**Figure 1:**
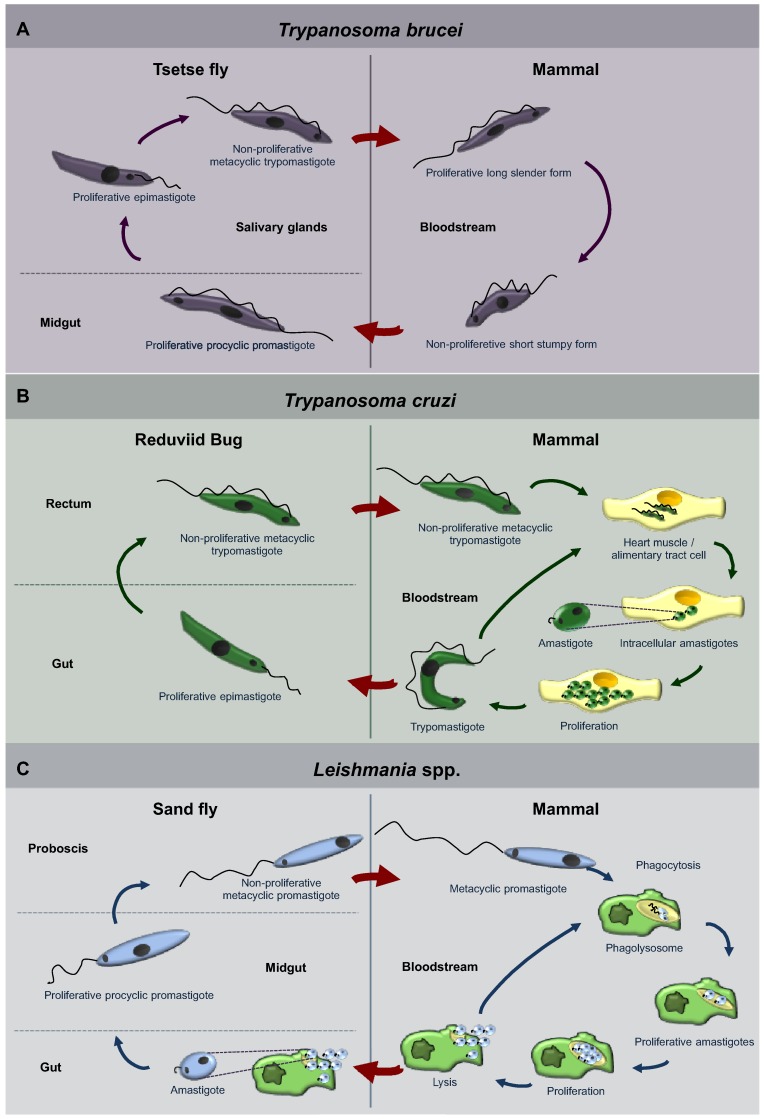
Diagrams of the life cycles of the three trypanosomatid parasites: *Trypanosoma brucei* (**A**), *Trypanosoma cruzi* (**B**), *Leishmania* spp. (**C**). Each of these parasites alternates between an insect host (left hand side of the panels) and a mammalian host (right-hand side). The body parts or cells in which the parasites reside are indicated, as well as the names of the different morphological forms that the parasites display during their successive differentiation steps. For a detailed description, see the main text.

Trypanosomatids of some genera only occupy a single host, whilst others have life cycles involving a secondary host which may be a vertebrate, invertebrate or plant. Different species go through a range of diverse morphologies (usually at least two) at several stages of their life cycle. Typically, the promastigote and epimastigote forms are found in insects, whilst trypomastigote and amastigote forms are found in the animal hosts, the former in the bloodstream and the latter inside host cells [5,6,7,22]. The life cycles of the three best studied trypanosomatids, *T. brucei*, *T. cruzi* and *Leishmania* spp. are represented in Figure 1. *T. brucei* is an exclusively extracellular parasite. Its trypomastigote metacyclic form is introduced in the mammalian bloodstream by a tsetse fly, its invertebrate host, during a blood meal. Once in the blood, parasites proliferate as long-slender forms. However, as parasite density increases they differentiate to so-called short-stumpy forms. Stumpy forms do not divide and are pre‑adapted for survival and differentiation when taken up by a tsetse fly (Figure 1A) [23]. On the other hand, *T. cruzi* and *Leishmania* are intracellular parasites during most of the time they spend in their mammalian hosts. *T. cruzi* is introduced as a metacyclic trypomastigote form in skin lesions and mucous tissues of the mammalian host by the feces of reduviid bugs that defecate during feeding. Trypomastigotes spread through the blood, so infecting any tissue, especially tissues from heart and the alimentary tract, where they develop into intracellular amastigotes that proliferate and finally differentiate to trypomastigotes. These latter forms are released in the blood and extend the infection by infecting new cells, or can be taken up by a reduviig bug (Figure 1B) [24]. On the other hand, *Leishmania* procyclic promastigotes are transmitted by a female phlebotomine sand fly when feeding on mammalian hosts. Once in the blood, promastigotes are phagocytosed and settle inside a phagolysosome, where they differentiate into amastigotes. Amastigotes proliferate, extending the infection, and the ones present in skin macrophages can be taken up by sand flies (Figure 1C) [25].

During their developmental cycles, trypanosomatids face very different conditions of pH, temperature, nutrients and oxygen supply. Consequently, the parasites’ metabolism differs in the different life-cycle stages, requiring an important metabolic reprogramming. In that aspect, autophagy is expected to play an important role.

### 1.2. General Aspects of Autophagy

**Figure 2:**
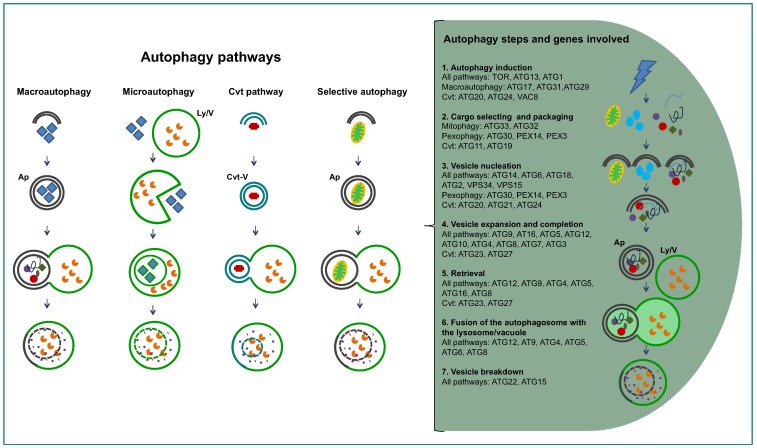
Diagram presenting the different autophagy-related pathways in yeast, and the successive steps of the pathways. On the right hand side, the successive steps are specified in text and cartoons, showing the proteins involved. Abbreviations: Ap, autophagosome; Cvt, cytosol to vacuole targeting; Ly/V, lysosome/vacuole.

The word autophagy was coined by Christian de Duve in 1963, to define the process by which the cell’s can degrade its own proteins [15]. Previously, de Duve had discovered the presence of lysosomes in mammalian cells, organelles with an acidic matrix containing a large number of hydrolases responsible for intracellular degradation of proteins and other macromolecules [26]. However, most of our current knowledge about autophagy has been acquired in the last decade, particularly by intensive genetic, molecular and cellular research carried out in the yeast *Saccharomyces cerevisiae*. More than 30 ATG (AuTophaGy-related) genes have been identified and, for many of them, molecular and functional studies have been performed, facilitated by the easy manipulation of these organisms [27,28,29]. At present, we know that there are several autophagic processes (Figure 2), macroautophagy being the most common one [30,31,32]. Macroautophagy is a non-selective process in which ATGs concentrate at the so-called PAS or Pre-Autophagosomal Structure. An isolation membrane is formed randomly around portions of cytoplasm, bulk cytosol and other cytoplasmic components such as organelles. The isolation membrane expands and closes to form a double-membrane vesicle, the autophagosome, which ultimately fuses with the lysosome, or with the vacuolar membrane in yeast, forming the autolysosome. The single membrane vesicle that is found inside the lysosome is termed autophagic body. On the other hand, another kind of autophagy, microautophagy, happens when lysosomes directly engulf cytoplasm by invagination, protrusion, and/or septation of the lysosomal limiting membrane. There are also some processes related to macroautophagy, for selective routing of macromolecules or organelles to lysosomes/vacuoles. The best studied of these latter processes is the cytoplasm-to-vacuole targeting (Cvt) pathway, identified only in the yeasts *S. cerevisiae* and *Pichia pastoris*, where in nutrient-rich conditions specific hydrolytic enzymes are routed, by use of dedicated vesicles, to their final destination, the vacuole [33]. The best-described protein to take this route is pre-aminopeptidase, that oligomerizes into dodecamers while in the cytosol and after delivery to the vacuole—in nutrient rich conditions by Cvt, in starvation conditions by macroautophagy—it is processed into its mature form. Other selective autophagy-like processes operate in order to degrade redundant or damaged organelles; examples are pexophagy (degradation of peroxisomes, that can take place by macro- or microautophagy, being termed macro or micropexophagy) [34], or mitophagy (partial or total mitochondrial degradation) [35], piecemeal microautophagy of the nucleus (PMN) [36] among others. The removal of redundant or damaged organelles is one of the functions of autophagy, but many other functions have been attributed to this process: removal of damaged cells, survival mechanism under starvation by recycling cytoplasmic constituents, assisting cellular survival under other stress conditions such as hypoxia and high temperatures, remodeling the cell’s morphology or/and metabolic machinery during nutritional changes or developmental differentiation, and even autophagy-dependent cell death [37,38,39,40].

Another mechanism participating in the recycling of intracellular proteins is the proteasome system. This system, conserved in all eukaryotes, drives mainly misfolded and aggregated cytosolic proteins marked with one or several ubiquitin moieties to a multiprotein complex, the proteasome, where proteolysis occurs. Protein modification by ubiquitin does however not serve exclusively for targeting proteins for proteasomal degradation. This signal also participates in cellular trafficking, immune response, DNA repair and chromatin remodeling [41]. This modification is also able to direct whole organelles for degradation in the lysosome, via autophagy [42,43].

In the last decade it has been shown that lysosomes and autophagy are present in a large variety of eukaryotes, including trypanosomatids [42,44,45]. In this review, we summarize the current knowledge of autophagy in trypanosomatids by describing the findings achieved at the molecular level as well as evolutionary aspects, and the role played by autophagy in the survival, life-cycle differentiation and pathogenicity of these parasites. Moreover, we discuss the possibility for chemotherapeutic interference to combat the diseases caused by them.

## 2. Inventory of Genes Encoding Proteins Involved in Autophagy in Trypanosomatids

Irrespective of the conditions that cause the need for degradation of cytoplasmic components, the autophagy pathway in any cell follows a similar series of steps [46] from its induction to the delivery of the target to the degradative organelle (Figure 2). The first such step is autophagy induction, where ATGs concentrate and interact at the PAS. The interactions between ATGs prime the *de novo* formation of the double-layered isolation membrane at the vesicle nucleation step. The vesicle expansion step is next, where different ATGs participate to bring lipids for increasing the membrane’s size and curvature. The closure of the isolation membrane causing the separation of its contents from the cytosol comprises the vesicle completion step. The newly formed vesicle is the so-called autophagosome. Next, the fusion of the outer membrane of the autophagosome with the lysosome/vesicle is followed by the degradation of the single membrane autophagic body and release of its contents into the lysosome/vesicle.

The complex comprising ATG1-ATG13-ATG17 in yeasts and ULK1-ATG13-FIP200-ATG101 in mammalian cells is involved in autophagy induction [47]. It is upstream of the complex centred on VPS34 (composed also by VPS15, ATG6/VPS30 and ATG14) [48] and the transmembrane protein ATG9, that participates in the membrane transport to the forming phagophore [49]. The elongation step requires two ubiquitin-like conjugation systems, one involving the E1-like ATG7 and the E2-like protein ATG10 to bind ATG12 to ATG5, that will form a complex with ATG16 [50]. The second one is centred around ATG8/LC3 and comprises the peptidase ATG4 along with ATG7 and ATG3 that function as an E1 and E2 protein, respectively. This complex serves to bind ATG8 to a phosphatidylethanolamine (PE) moiety in the expanding phagophore. The ATG12-ATG5-ATG16 complex seems to serve as an E3-like protein and increases the rate of the association of ATG8 to the membrane [51]. ATG8 is a widely used autophagy marker [52], being the only ATG that remains attached to the autophagosomal membrane and can be delivered to the vacuole/lysosome, while other ATGs disperse from the PAS.

The trypanosomatids genome databases and the range of ATGs identified from yeast species have been used to predict if the parasites possess a conserved autophagy pathway. An initial BLAST search using 40 ATGs and other proteins involved in autophagy from *S. cerevisiae* has identified in trypanosomatids homologues for about 20 of these gene products that participate in each of the steps of the pathway in yeast [53]. While an indication that autophagy takes place in these parasites, the limited number of proteins identified seemed to point to a less complex autophagy pathway than what is observed in yeasts. Or alternatively, some different, still unknown proteins, not homologous to yeast ATGs, may be involved in the trypanosomatids. A second search was done in 2009 [54], where more recently identified yeast ATGs such as ATG25, ATG28 and ATG30 were also used. And more recently it has been reported that there are no detectable homologues for ATG29, ATG32 or ATG33 [42]. A summary of the candidate ATGs, and homologues of other yeast proteins involved in autophagic processes identified in the genomes of the trypanosomatid parasites, is presented in Table 1. Moreover, the Table indicates which of these proteins have been functionally characterized in recent research, confirming or not their role in autophagy-related pathways in the trypanosomatids.

One obstacle for the characterisation of the complex involved in autophagy induction in the parasites is the fact that trypanosomatids possess too many possible homologues for the kinase ATG1. Upstream acting TOR1 and TOR2 homologues were readily identified in all three trypanosomatids. *T. brucei* possesses one copy for TOR1 and TOR2 as well as a TOR-like 1 and a TOR-like 2 gene [55], while *L. major* has a TOR-1, a TOR-2 and a TOR-3 [56], with the latter being similar to TOR-like 1 from *T. brucei*. *T. cruzi* has a TOR1 and a TOR2.

The class III phosphatidylinositol 3-kinase (PI3K) VPS34 and its interacting partners, that participate in the vesicle nucleation step (ATG6/VPS30 and VPS15), possess homologues in the trypanosomatid genomes, however ATG14 and VPS38, responsible for the specificity of the complex between autophagy and ‘vacuolar protein sorting’, seem to be absent.

For the autophagosome marker ATG8, more than one gene were identified for each of the trypanosomatids: three for *T. brucei*, two for *T. cruzi* and, surprisingly, four families comprising together 25 genes for *Leishmania major*. *T. brucei* has two very similar ATG8s (ATG8.1 and ATG8.2) and a more distant ATG8.3. *T. cruzi* has a ‘true’ ATG8.1 and an ATG8.2 that does not seem to participate in autophagy [57].

**Table 1:** Proteins predicted—by genomic searches—or proved to be involved in the successive steps of autophagy (see Figure 2) in trypanosomatids. In blue: proteins for which orthologues were found in the trypanosomatid genome databases. In green: proteins for which homologues (not orthologues) could be found in the trypanosomatid genomes. In red: proteins for which no orthologues or homologues were identified in the trypanosomatid genomes. One asterisk indicates proteins whose role in autophagy in trypanosomatids has been proved. Two asterisks indicate proteins that have been characterized in trypanosomatids, but their role in autophagy has not yet been determined. Details of the genomic searches, performed with known yeast and mammalian autophagy components, have been described previously [42,53,54,58].

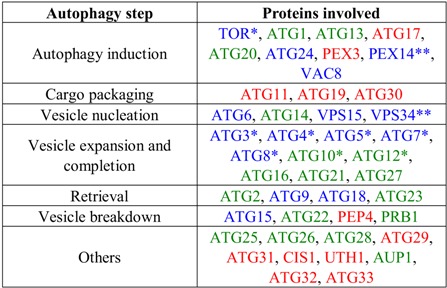

Unlike yeast, where one ATG8 and one ATG4 are found, and more similar to mammals having several homologues of ATG8 with apparently different roles in the cell, trypanosomatids possess not only multiple ATG8 homologues but also two paralogues for ATG4. Different specificities and efficiencies have been described for *L. major* [59] and *T. cruzi* [57] ATG4s (see next session).

The ubiquitin-like conjugation system involving ATG8 is present in all three trypanosomatids described here, while none of the components of the ATG12 conjugation system could initially be identified [53]. However, later studies identified and functionally characterized ATG12, ATG5 and ATG10 in *L. major* [59]. It appears that the ATGs identified as TbATG8.3 and TcATG8.2 by sequence and phylogenetic analysis are syntenic with LmATG12, and therefore may perform the ATG12 function in the *Trypanosoma* species.

Moreover, homologues for the genes of general autophagy-related proteins ATG13, ATG14, ATG17, ATG22, ATG23 and ATG27, and of the Cvt pathway-specific ATG11, ATG19, ATG20 and ATG21 were not found in the trypanosomatids, nor genes for the yeast mitophagy-specific UTH1 and AUP1 and pexophagy-specific ATG11, ATG25, ATG28 and ATG30. While the Cvt pathway seems restricted to fungi, the absence of several specific autophagy homologues in the trypanosomatid genomes should not be taken as an indication that there is no specific autophagy taking place in these cells, as it is known from studies with yeasts that specific-autophagy genes such as ATG28 and ATG30 in *P. pastoris* or UTH1 and ATG29 in *S. cerevisiae* (this latter also found in *Yarrowia lipolytica*) are often found to be exclusive to one species or its closest relatives [30]. From the retrieval and vesicle breakdown steps of autophagy, few of the proteins have homologues in the trypanosomatids. This is the case for ATG9, ATG18 and the peptidase ATG15, while ATG2, ATG22 and PEP4 were not found either. Furthermore, for most of the trypanosomatid proteins identified by the genomic searches, the role in autophagy needs still to be proved by experimental work (Table 1), while it remains to be determined if other proteins are present that exert in the trypanosomatids the function of the missing yeast homologues. While the key autophagy protein, ATG8, of trypanosomatids has already received considerable attention for research [59,60], with the crystal structure of TbATG8.2 even been solved [61], other important proteins are still to be identified, such as ATG1. And still others have been characterized but their role in autophagy has not yet been shown, as is the case for VPS34 [62]. Surely, we may expect more autophagy-related proteins to be identified in the trypanosomatids, and it is likely that future research will be able to associate already known proteins to this pathway.

## 3. Experimental Studies on Autophagy in Trypanosomatids

As described in Section 1.1 of this review, most of the trypanosomatids have a life cycle in which they alternate between an insect and a mammalian host, requiring profound metabolic and morphological changes as adaptations to the different environments encountered. It was suggested that autophagy may play a fundamental role in these developmental changes [53,58]. The first morphological indications that autophagy occurs during differentiation of trypanosomatids were provided by electron microscopy images of *T. brucei* taken by Vickerman and colleagues in the 1970s [63]. However, this aspect of their impressive morphological analysis of the trypanosome during its developmental cycle received little attention. It was only in the last few years, after the discovery of ATG homologues in trypanosomatids, that extensive research on autophagy was performed and the process was experimentally demonstrated to occur in these parasites and that different aspects of it were analyzed. We will proceed by describing the experimental studies about autophagy that have been carried out in the three most studied trypanosomatids.

### 3.1. Autophagy in T. brucei

The bloodstream form of *T. brucei* lives in an environment where glucose is present at a reasonably constant level. Consequently, and because the parasite’s single mitochondrion is highly repressed at this stage, bloodstream forms obtain all their energy by generating ATP via glycolysis. The majority of the enzymes of the glycolytic pathway are uniquely sequestered in peroxisome-like organelles which were hence called glycosomes [8,64]. This compartmentalization is essential for the viability of the bloodstream form; disruption of the biogenesis of the organelles, causing mislocalization of newly synthesized glycolytic enzymes to the cytosol, has been shown to be highly deleterious, eventually leading to death of the parasites [65,66]. This was attributed to an unregulated activity of the relocated enzymes with as result an accumulation of glycolytic intermediates to very high, apparently toxic levels [10,11,67]. By contrast, the procyclic form of the parasite presents a more complex metabolism which involves a well developed mitochondrion with functional Krebs cycle enzymes, respiratory chain and oxidative phosphorylation enzymes. This form relies more on the oxidation of amino acids like proline, abundantly present in the midgut of the tsetse fly, while glucose is present only occasionally for a short time, after the fly has taken a bloodmeal [9]. In agreement with these changes, the enzyme content of glycosomes changes considerably, with a down-regulation of glycolysis and an upregulation of several other pathways [8,9].

**Figure 3:**
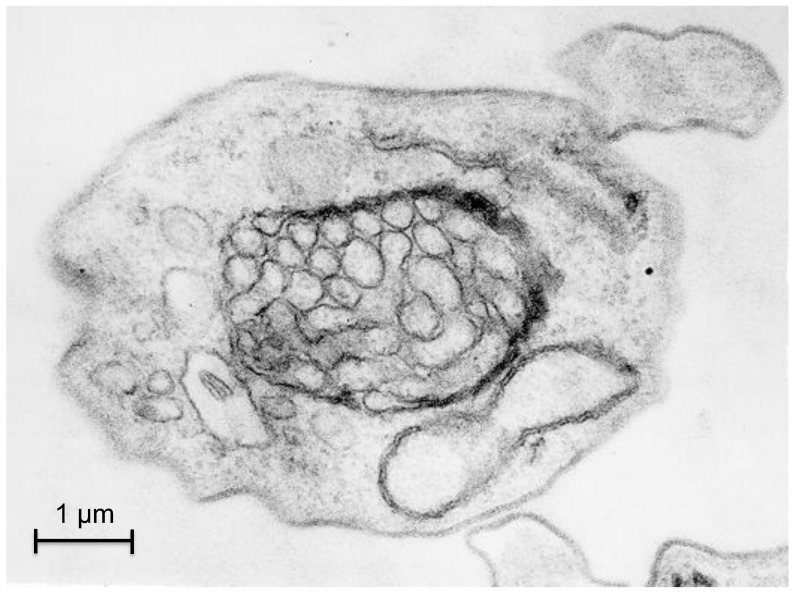
Pexophagy in *Trypanosoma brucei*. The picture represents a transmission electron micrograph of a cross section of a trypanosome showing an autophagosome-like, double-membrane structure containing multiple glycosomes.

Strong indications have been obtained that autophagy participates in this remodeling of the number, size and enzymatic contents of the glycosomes during the development of the parasite. Electron microscopy pictures confirm that glycosomes can be sequestered within double-membranes of autophagosome-like structures (Figure 3). Moreover, results obtained by Herman and colleagues [68] suggested an efficient glycosome turnover involving autophagy in *T. brucei*, which seems reminiscent of the situation observed for specific autophagy of peroxisomes (pexophagy) in methylotrophic yeasts [69]. A tendency of glycosomes to associate with the lysosome was observed when, in infected mice, a population of long-slender bloodstream forms differentiated into short-stumpy forms, which are pre-adapted to life in the tsetse fly. This tendency was dramatically enhanced during the short‑stumpy to procyclic transformation of *in vitro* cultured parasites. In this way, ‘old’, redundant glycosomes are degraded while new organelles become synthesized, containing an enzyme repertoire better adapted to the lifestyle of the new form of the parasite. The fact that the lysosome shows a transient but dramatic increase in size during the differentiation process could suggest that macropexophagy would be involved in the formation of autophagosomes. However, glycosomes seem to accumulate at the lysosome and become surrounded by it, suggesting quite the opposite, a process similar to micropexophagy [68]. This latter observation is supported by a bioinformatics analysis, since orthologues of most of the ATG proteins that are specifically involved in micropexophagy in *S. cerevisiae* are present in *T. brucei* [53]. This seems to contrast with the situation for *Leishmania* species, where only macroautophagy has been experimentally observed so far, as it will be described below [70,71].

The typical marker used for the study of autophagy in many organisms is ATG8. As already mentioned, African trypanosomes possess three ATG8 isoforms: two which are more closely related to human ATG8/LC3, *Tb*ATG8.1 and *Tb*ATG8.2, and one called *Tb*ATG8.3. This latter one is, based on phylogenetic criteria, a typical ATG8, but its gene is syntenic to that of a protein which in *L. major* has been shown to exert the ATG12 function (see below). Punctate structures proposed to be autophagosomes were detected by the expression of a fusion construct of yellow-fluorescent protein and *Tb*ATG8.2 in procyclic cells exposed to endoplasmic reticulum (ER) stress by DTT treatment. However, these autophagosomes appeared in cells that also expressed apoptotic markers, resembling the apoptosis process that takes place in mammalian cells under persistent ER stress [72]. Furthermore, the crystal structure of *Tb*ATG8.2 has been obtained, showing the classic ubiquitin fold [61]. This, together with a high sequence identity, strongly suggests that *Tb*ATG8.2 is the functional homologue of the human ATG8/LC3. Upon starvation or upon induction of differentiation, both *Tb*ATG8.1 and *Tb*ATG8.2 concentrate in *T. brucei* into punctate structures reminiscent of autophagosomes [60,73]. However, *Tb*ATG8.1 and *Tb*ATG8.2 appear not to be fully equivalent. Depletion of *Tb*ATG8.1 does not cause detectable effects on *Tb*ATG8.2 recruitment to autophagosomes, whereas *Tb*Atg8.2 depletion significantly reduces the autophagosome relocation of *Tb*Atg8.1. Furthermore, Li *et al*. [60] described that depletion of *Tb*ATG8.1 and *Tb*ATG 8.2, individually or together, promotes cell survival under starvation conditions. Unexpectedly, their results seem to indicate that autophagy in trypanosomes is detrimental when the cells are brought under starvation conditions. *T. brucei* also contains two ATG4 homologues, *Tb*ATG4.1 and *Tb*ATG4.2. When docking *Tb*ATG8.2 to a homology model of *Tb*ATG4.1 (the one with higher sequence identity with human ATG4), the overall structure seems very much alike and the necessary catalytic triad (C-D-H) is well conserved in *Tb*ATG4.1, strongly suggesting conservation of function [61].

In higher eukaryotes, the balance between protein synthesis and degradation via autophagy is regulated by TOR, a well-studied kinase that is mostly known, and received its name for being the target of the antibiotic rapamycin. There are two different TOR-containing complexes in all the eukaryotes studied, with capabilities of governing spatial and temporal cell growth separately. Generally, complex 1 (TORC1) controls temporal aspects of cell growth through processes such as ribosome biogenesis, transcription, translation and repression of autophagy, while TORC2 controls spatial aspects of cell growth by actin cytoskeleton remodeling [74]. In animals, there is just a single TOR kinase, mTOR, which participates in both complexes. However, two TOR kinases, TOR1 and TOR2, are found in single-cell organisms [74,75]. TOR1 and TOR2 are also present in *T. brucei* (*Tb*TOR1 and *Tb*TOR2), where they bind to and control signaling through *Tb*TORC1 and *Tb*TORC2, respectively [55,76]. As in yeast, both kinases are associated with the orthologues of TORC1 and TORC2 partners, KOG1/raptor and AVO3/rictor. *Tb*TOR1 is mainly located in the nucleus, whereas *Tb*TOR2 is excluded from the nucleus and is associated with the ER and the mitochondrion. In addition, trypanosomatids present two more TOR-related proteins, *Tb*TOR-like 1 and *Tb*TOR-like 2, that do not seem to form part of the TOR complexes. As in other eukaryotes, *Tb*TOR2 seems to regulate actin polarization towards the endocytic pathway, playing a crucial role in endocytosis and cytokinesis, while the *Tb*TOR1 function is to promote cell growth and proliferation by positively regulating protein synthesis, cell size, cell cycle progression and RNA polymerase I localization to the nucleolus. However, when bloodstream forms of *T. brucei* were treated with rapamycin, there was a pronounced reduction of cell proliferation but, contrary to what occurs in other eukaryotes, the effect was caused by inhibition of TORC2, rather than TORC1. Used in the nanomolar range, rapamycin inhibits *Tb*TORC2, producing actin depolarization which interferes with endocytosis and cytokinesis. Moreover, a rapamycin-FKBP12 complex (FKBP12 is the mediator that lets rapamycin to bind TOR) does not bind to the recombinant *Tb*TOR1 rapamycin-binding domain, while it does to *Tb*TOR2 both *in vitro* and *in vivo*. Taken together, these experiments suggest that rapamycin cannot be used for triggering the autophagic process in *T. brucei*, because *Tb*TOR1 seems to be rapamycin-insensitive [55]. Nonetheless, it has been shown that rapamycin is able to induce growth inhibition leading to cell death of bloodstream-form *T. brucei* (apparently different from apoptosis and necrosis) with the formation of structures that are reminiscent to phagophores, autophagosomes and autolysosomes. However, in order to observe this effect, a concentration 10-fold higher than that used to induce autophagy in yeast was required [77]. Moreover, and although trypanosome TORC1 is insensitive to rapamycin, it was shown by RNAi that *Tb*TOR1 knockdown triggered the appearance of autophagic-like vesicles. Its depletion also caused morphological changes such as abnormal appearance of the ER and formation of multi-lamellar membranes, and it conferred partial resistance to stress situations, as it happens in other eukaryotes when TOR1 is inhibited [76].

The possibility of using trypanosomatid TOR enzymes as drug targets received recently further support by the demonstration that *Trypanosoma* and *Leishmania* species were highly susceptible for mammalian mTOR and PI3K inhibitors that are currently in pre-clinical and clinical development for cancer treatment [78]. Several inhibitors exerted growth inhibition of cultured parasites at micromolar or better efficacy. One compound was effective at sub-nanomolar concentrations and cleared parasitaemia in a *T. brucei* infected animal model. Although TOR as the *in vivo* target of these compounds in the parasites remains to be proved, the results are highly promising. Moreover, the large sequence difference of the human and trypanosome enzymes offer promise for increasing the selectivity of the inhibitory compounds.

In several cases it has been shown that the process of autophagy can also be induced in African trypanosomes by the addition of different molecules to the medium. One of these molecules is dihydroxyacetone (DHA) [79,80]. Whilst many cells possess a DHA kinase to phosphorylate the DHA, to feed it into the glycolytic pathway, trypanosomes, which take this molecule up via aquaglyceroporins, cannot metabolize DHA since they lack the DHA kinase. This non-metabolizable form is toxic; DHA causes growth inhibition of *T. brucei* bloodstream forms (with an EC_50_ of 1 mM) as a result of cell cycle arrest in the G2/M phase, as well as morphological alterations such as an increase of vesicular structures within the cytosol and the presence of multivesicular bodies, myelin-like structures, autophagy-like vacuoles and a marked disorder of the characteristic mitochondrion structure. Furthermore, Delgado and colleagues reported that some neuropeptides can be endocytosed by *T. brucei* bloodstream forms, inducing autophagy in the parasites [81,82]. The authors showed that endocytosed peptides reach the lysosome, whose integrity is subsequently affected. Moreover, the peptides can be released from the late endosomes or lysosome and then accumulate in various intracellular compartments, causing partial relocalization of glycosomal enzymes. This provokes a failure of the energy metabolism that results in cell death, with no signs of necrosis or apoptosis. The presence of autophagosomes in these neuropeptides-treated parasites is supported by immunostaining using mammalian anti-ATG8/LC3 antibodies [81,82].

### 3.2. Autophagy in Trypanosoma cruzi

It has been described that the process of differentiation of *T. cruzi* epimastigotes into metacyclic infective forms that occurs in the insect's rectum may be triggered by the stress of nutrients limitation. The insect’s epimastigote stage contains a lysosome-like organelle designated the reservosome, that stores endocytosed material, lipids and secretory proteins, as well as a major cysteine proteinase, cruzipain [83]. Apparently, massive proteolysis occurs during the differentiation to metacyclic trypomastigotes that can be reproduced *in vitro* by inducing starvation of cultured epimastigotes. Cruzipain seems to be the responsible for this proteolysis, as inferred from the fact that cysteine proteinase inhibitors can block the differentiation [84,85].

The role played by the TOR kinases in autophagy (or any other pathway) in *T. cruzi* has not yet been addressed. Similarly for *Tc*VPS34, whose role in autophagy remains to be elucidated. However, this class III PI3K has been shown to be involved in osmoregulation and receptor-mediated endocytosis [86].

As mentioned above, *T. cruzi* contains two ATG8 homologues, *Tc*ATG8.1 and *Tc*ATG8.2. It was observed that differentiating and starved epimastigotes show an intense *Tc*ATG8.1 staining. In contrast, almost no signal was found in metacyclic trypomastigotes, and the weakly visible *Tc*ATG8.1 co-localized in part with the carboxypeptidase located in reservosomes. This suggests the delivery of the autophagosomal content to the lysosome system, which occurs as one of the final steps of the autophagy process [57].

*T. cruzi* also contains two ATG4 isoforms, *Tc*ATG4.1 and *Tc*ATG4.2. In an *in vitro* cleavage assay, *Tc*ATG4.1 was able to rapidly process both *Tc*ATG8.1 and *Tc*ATG8.2, while *Tc*ATG4.2 showed little activity towards these proteins. However, both *Tc*ATG4 genes could complement a yeast ATG4 mutant. On the other hand, only *Tc*ATG8.1 could partially replace the function of yeast ATG8, while *Tc*ATG8.2 could not. Moreover, only HA-tagged *Tc*ATG8.1 localized to large vesicles that were thought to correspond with autophagosomes. This localization appeared to be dependent on the key glycine residue at the C-terminus of the protein, since mislocalization occurred when the residue had been mutated. By contrast, HA-tagged *Tc*ATG8.2 only showed a weak punctate localization during starvation, and its function still remains unknown [57]. Taking together, these results clearly suggest that *Tc*ATG8.1 is the functional homologue of yeast and mammalian ATG8.

As in *T. brucei*, several molecules have been described to induce autophagy in *T. cruzi*. Three protein kinase inhibitors, staurosporine (a serine/threonine kinase inhibitor), genistein (a tyrosine kinase inhibitor) and the PI3K inhibitor wortmannin were able to cause antiproliferative effects in *T. cruzi* epimastigotes with the production of autophagosomes, among other ultrastructural changes. However, these drugs did not interfere with the division of intracellular amastigotes or with their differentiation to trypomastigotes [87]. Other compounds like edelfosine (a lysophospholipide analogue) or ketoconazole (an ergosterol biosynthesis inhibitor) induced morphological alterations in the plasma membrane and in reservosomes of epimastigotes, when used independently. Used in combination, they also led to severe mitochondrial damage, formation of autophagic structures and multinucleation [87]. Other trypanocidal agents, such as the dinitroaniline herbicide trifluralin and its intermediate chloralin, induced the appearance of vacuoles containing damaged membranes [88]. Furthermore, epimastigotes and trypomastigotes can also be affected by the action of some natural products. Geranylgeraniol, an oleaginous ethanolic extract of *Pterodon pubescens*, caused the appearance of concentric membrane structures in the cytosol associated to the autophagy process. Propolis, a resinous material collected by honeybees from different plant exudates, led to reservosome disorganization in epimastigotes. Naphthoquinones derived from β-lapachone (obtained of *Tabebuia* sp. trees) have been shown to cause atypical membrane structures, suggestive of the involvement of autophagy [88]. More recently, it has been demonstrated that a novel triazolic naphthofuranquinoneinduced many autophagy-related anomalies in the different forms of the parasite, as well as an impairment of the mitotic process [89]. Finally, three naphtoimidazoles also derived from β-lapachone affected the reservosome in epimastigotes and trypomastigotes, as well as the mitochondrion and Golgi, produced concentric membrane structures, and resulted in overexpression of several ATG genes and an increase of the number of autophagosome-like bodies, ultimately causing death of the parasites [90,91,92]. This effect is inhibited by E64 and the calpain I inhibitor, suggesting the involvement of calpain in the autophagic process [91] as it happens in mammals [93]. The death observed seems to be caused by the autophagic process induced by naphtoimidazoles, because the autophagy inhibitors wortmannin and 3-methyladenine are able to completely abolish the effects [90].

### 3.3. Autophagy in Leishmania spp.

As trypanosomes, the multiple species that belong to the genus *Leishmania* also face very different conditions during their life cycle. *Leishmania* promastigotes living in the alimentary tract of a sand fly are exposed to a temperature of around 26 °C and rather basic conditions, whereas the amastigotes present within the phagolysosome of mammalian macrophages are exposed to a higher temperature, an acidic pH, low levels of oxygen and different nutrimental conditions [94]. The combination of elevated temperature and acidic pH stress has been recently reported to act as a key signal triggering the promastigote to amastigote differentiation. This signal leads to a marked decrease in global translation initiation associated with eIF2a phosphorylation, while translation of amastigote-specific transcripts such as the major virulence factor A2 is preferentially upregulated [95].

However, and contrary to what has been described for *T. brucei*, the available data suggest that the metabolic reprogramming required during differentiation of promastigotes to amastigotes does not involve increased glycosome turnover by pexophagy. The altered metabolic fluxes seem rather the result from different nutrient availability and posttranslational regulation of changes in enzyme activities, than from the introduction of major changes in the metabolic enzyme repertoire [96,97]. In *Leishmania*, pexophagy may be more involved in an adjustment of the number of glycosomes, associated with the dramatic decrease of cell size during this differentiation step, instead of a switch of the glycosomal enzyme content [98,99].

On the other hand, it has been shown that protein turnover by macroautophagy is essential for metacyclogenesis (the differentiation of procyclic promastigotes into highly infective metacyclic promastigotes) and also for the differentiation of metacyclic promastigotes into amastigotes, as reviewed by Besteiro and colleagues [94]. In the different *Leishmania* species, the lysosome presents multiple shapes which reflect the distinct functions in the various developmental forms [100]. While promastigotes contain a tubular-vesicular compartment termed the multi-vesicular tubule (MVT) that accumulates FM4-64 and other endocytosed markers and contains lysosomal cysteine and serine peptidases, amastigotes are characterized by the presence of a large membrane-bounded compartment, the megasome, which has an acidic pH and contains many peptidases. These lysosome-like structures have highly varying enzyme contents depending on the developmental stage of the parasite, with especially the peptidase activity being upregulated during metacyclogenesis. In fact, two lysosomal cysteine peptidases (CP), CPA and CPB, have been directly implicated in both autophagy and development. Their expression increases during differentiation together with the appearance of megasomes. Moreover, promastigote mutants lacking these enzymes have an enhanced number of autophagosome-containing MVTs. Although the deletion of both proteins is not deleterious, the differentiation into amastigotes appears to be largely prevented and cells are not able to degrade Green Fluorescent Protein (GFP)-ATG8 labeled autophagosomes within the lysosomes. This seems to indicate that both peptidases fulfill the function that in yeast is exerted by the two major peptidases, PEO4 and PRB1, which are absent in the *Leishmania* genome [71]. Moreover, the blocking of endosomal sorting at a late stage by inactivation of the *L. major VPS4* gene resulted in an accumulation of cytosolic autophagosomes that could not be processed further in the MVT-lysosome. VPS4 is an ATPase involved in the endosomal system and in the formation of late endosomes with a multivesicular aspect called multivesicular bodies (MVB). As a consequence of VPS4 inactivation, the ability of the parasites to survive under nutrient deprivation conditions was affected and they were unable to differentiate into metacyclic promastigotes. In addition, a mutant of the ATG4.2 cysteine peptidase from *L. major* was also found to be defective in the ability to differentiate, supporting a role of autophagy during the differentiation process. Although in this mutant GFP-ATG8 puncta were still present during starvation, highly increased levels of the lipidated form of ATG8 were observed [70]. Moreover, *in vitro* experiments showed that *Lm*ATG4.1 presents higher proteolytic activity towards *Lm*ATG8 than *Lm*ATG4.2 does [59]. Taken together, these results suggest that the *LmATG4.2* mutation affects the release of *Lm*ATG8 from PE at the autophagosomal membrane, rather than the activation of *Lm*ATG8 prior to its conjugation to PE.

As mentioned above, four families of ATG8 were found in *Leishmania*: ATG8, ATG8.A, ATG8.B and ATG8.C. Together, the different families are able to fulfill the action of the unique ATG8 from yeast [59], but only *Lm*ATG8 shows a high similarity to yeast and mammalian ATG8, and has been successfully used to label autophagosomes [70]. *Lm*ATG8.A also appears to localize to autophagosomes in promastigotes, but participating only in starvation-induced autophagy, while *Lm*ATG8.B and *Lm*ATG8.C seem not to be involved in autophagy [59]. Curiously, and in addition to the four ATG8 families, *L. major* also presents an ATG8-like protein with a C-terminal extension after the key glycine residue that is essential for conjugation to ATG5. Although this protein clusters with ATG8s in a phylogenetic analysis, it was named *Lm*ATG12 because it contains a 58-residue insertion that makes it quite similar to yeast and mammal ATG12, and indeed behaved as an ATG12 in yeast complementation studies. *Lm*ATG4 proteins are unable to cleave the C-terminal extension from *Lm*ATG12, but since only a mutated *Lm*ATG12 with a free C-terminal glycine can rescue a yeast *ATG12* null mutant, it is thought that an alternative peptidase must be present in order to free the glycine residue. *L. major* homologues of ATG5 and ATG10, despite their weak similarity, could also replace their yeast counterparts [59]. *Lm*ATG12 partially co-localizes with GFP-*Lm*ATG8 to punctate structures in starving *L. major* promastigotes. Additionally, Williams and colleagues have recently demonstrated the presence of a functional ATG12-ATG5 conjugation system in *L. major*, which is required for ATG8-dependent autophagosome formation. A null mutant of ATG5 and in consequence the lack of autophagy, leads to perturbation of the phospholipid balance in the mitochondrion, possibly through ablation of membrane use and conjugation of mitochondrial PE to ATG8 for autophagosome biogenesis, resulting in a dysfunctional mitochondrion with impaired oxidative ability and energy generation that causes a decrease in the virulence of the parasites [101].

The autophagy inducing proteins, *Lm*TOR1 and *Lm*TOR2 are essential proteins and have not been characterized yet, however *Lm*TOR3 has been shown to be involved in acidocalcisome biogenesis [56]. Recently, a protein kinase A regulatory subunit of cAMP-dependent protein kinase (Ldpkar1) was described to play an important role in metacyclogenesis in *L. donovani* by interfering with the autophagy pathway. Starvation conditions cause the overexpression of the protein, which leads to an acceleration of autophagy. Consistently, low levels of Ldpkar1 delay the induction of autophagy in the parasite [102].

Several compounds have been also shown to cause ultrastructural alterations in *Leishmania* that can be related to autophagy. The aziridine-2,3-dicarboxylate-based cysteine cathepsin inhibitor 13b (derived from natural aziridinyl peptide miraziridine A isolated from the marine sponge *Theonella* sp.) induced cell death in *L. major* promastigotes. Accumulation of debris in autophagy-related lysosome-like vacuoles was observed at an early phase of the dying of the parasites, but death seemed to finally occur by an apoptosis-like death mechanism [103]. *L. chagasi* and *L. amazonensis* promastigotes treated with yangambin, a lignan obtained from *Ocotea duckei*, also showed features of both apoptosis and autophagy [104].The sesquiterpene elatol, the major constituent of the Brazilian red seaweed *Laurencia dendroidea*, showed an antiproliferative effect against *L. amazonensis* promastigotes (at 4 μM) and amastigotes (at 0.45 μM), causing diverse changes including the appearance of concentric membrane structures and formation of membrane structures suggestive of an autophagic process [105]. More recently, it has been shown that *L. amazonensis* parasites treated with Amiodarone, a drug commonly used to treat arrhythmics, presented amongst many other alterations including severe mitochondrial swelling, the formation of large autophagosomes that contained part of the cytoplasm and membrane profiles [105]. Antimicrobial peptides were also able to cause the death of *L. donovani* promastigotes with the appearance of vacuoles that were stained with monodansylcadaverine, a biochemical marker of autophagy [106].

### 3.4. Roles of Autophagy in Trypanosomatids Inferred by Experimental Studies

The experimental evidence for autophagy in trypanosomatids presented above provides us with a quite realistic idea of the multiple roles that autophagy can play in these parasites. As we have seen, one of the most important roles of autophagy is related to the differentiation of the parasites during their life cycle [58]. Autophagy is involved in remodeling the cell’s morphology and/or the metabolic machinery by elimination of components that are not necessary for the new life-stage. It can also serve as a survival mechanism under starvation or other stress conditions such as hypoxia and high temperatures by recycling cytoplasm constituents. However, not only the autophagic process that occurs in the parasite is important for its successful life cycle, but—at least for some, notably intracellular parasites—also autophagy by cells of the mammalian host can be a key process, as it may favor the parasite’s colonization and/or virulence. When *T. cruzi* infects mammalian cells, a signal transduction cascade is activated that leads to the formation of its parasitophorous vacuole, which has been shown to be labeled by the host cell autophagic protein LC3. Furthermore, autolysosomes are recruited to parasite entry sites, and starvation or pharmacological induction of autophagy of mammalian cells significantly enhances the infection, whereas inhibitors of the autophagic pathway reduce the invasion. Moreover, the absence of Atg5 or the reduced expression of Beclin 1, two proteins required at the initial steps of autophagosome formation, limit parasite entry and reduce the association between the parasitophorous vacuole and the classical lysosomal marker Lamp-1 [107]. 

We have also mentioned several examples of how autophagy has been described to be a mechanism of cell death in trypanosomatids, as it has been previously described for other organisms. In some of these cases, the appearance of autophagosome-like bodies seems to be accompanied by the characteristic cellular integrity that corresponds with an autophagy process, where treated cells present a normal nucleus and show no alteration of the mitochondrion, nor permeabilization of the cellular membranes. However, in many cases the cell phenotype is characterized by a mix of structural changes that could correlate with both autophagy-related and apoptotic-like processes. A new link between autophagy and apoptosis has been recently discovered in mammals, showing that ATG12 can bind to anti-apoptotic Bcl-2 family members to promote apoptosis [108,109]. This supports the other reasons why doubts have been raised about the existence of ‘autophagic cell death’, based on the fact that extensive literature illustrates that autophagy may be involved in lethal signaling, although none of them formally establishes that autophagy itself is responsible for cell killing. Consequently, it has been suggested that autophagy usually constitutes a futile attempt of dying cells to adapt to lethal stress rather than a mechanism to execute a cell death program [40], although the issue remains highly debated [110]. Following this stream of thoughts, cryptolepine-induced cell death of *L. donovani* has been shown to result from the cells counteracting the toxic effects of the compound by eliciting initial autophagic features. Indicating that autophagy indeed serves as a survival mechanism in response to cryptolepine treatment in *L. donovani* promastigotes, is the fact that inhibition of autophagy causes an early increase in the number of cells that die [111]. Contrary to this, as already mentioned, it has been shown that depletion of TbATG8.1 and 8.2, individually or together, promotes cell survival under starvation conditions, suggesting a pro-death role for autophagy in *T. brucei* [60].

Proteasomes play also a role in protein recycling in addition to autophagy. This protein complex with protease activity that works independently of the lysosome, is conserved in all eukaryotes, including trypanosomatids. The *T. brucei* proteasome is the best-characterized one in any parasitic protist. Relevant for drug discovery is the fact that not only important differences exist between the proteasome components of trypanosomatids and mammalians, but also that the requirement of this complex for the proliferation and differentiation of the parasite has already been established for *T. brucei*, *T. cruzi* and *L. mexicana* [112,113]. Indeed, proteasome inhibitors are known to be trypanocidal [114].

## 4. Conclusions and Perspectives

The occurrence of autophagy in trypanosomatid parasites has been convincingly demonstrated, first by bioinformatics approaches, and subsequently experimentally in a variety of molecular and cellular studies. Previously, we have shown, on the basis of genomic searches, that autophagy is a ubiquitous eukaryotic process, because a canonical autophagy machinery is present in representatives of all major eukaryotic lineages thus must have already been present in the common ancestor [42,54]. However, comparative bioinformatics revealed that, on multiple occasions, the machinery has undergone moderation—elaboration or reduction, or even outright loss in many protists. In the trypanosomatids it has been retained, but homologues could be detected for only about half of the proteins that are involved in yeasts in the various autophagy pathways [42,53,54]. Whether this should be interpreted as the existence of a simplified process in these parasites, or if functions of yeasts ATGs are fulfilled by other proteins in these parasites remains to be determined. In this respect, it might be important to realize that the autophagy process in yeasts is highly specialized and that recently it has been argued that it may be considered as not being the most representative of the process in eukaryotes in general [115].

Autophagy in these parasites may—at a basal level—play a similar role as in other eukaryotes, *i.e.*, turnover of proteins to eliminate damaged cell constituents, such as proteins and organelles. However, a more important role is exerted in the remodeling of the morphology and metabolic capacity of the parasites during the various differentiation processes associated with the successive transitions in their complicated life cycle that involves different hosts and host compartments, exposing the parasites to highly different environments. Since these differentiation processes are essential for the parasites, their autophagy pathways may be a promising drug target against the serious tropical diseases caused by them and for which no adequate therapy is available. The low degree of sequence conservation of ATG proteins reinforces the notion that these proteins might be suitable targets for the development of trypanosomatid *versus* human protein selective inhibitors. Such compounds might then be used as leads for the development of drugs against diseases such as sleeping sickness, Chagas disease and leishmaniasis. In addition, considerable evidence is available that treatment of the parasites with a large variety of toxic compounds trigger autophagy-like processes. It is inferred that autophagy is used by the parasites in an attempt to cope with the stress caused by the compounds. Often the response is insufficient, and the parasites die. Some authors interpret this as ‘autophagy-related cell death’, but it remains to be established whether it should not rather be considered as ‘cell death despite autophagy’. In the latter case, combination therapy of drugs interfering with both metabolic processes and autophagy in the parasites may have an additive or even synergistic effect.

We are not aware of any study on the effects of the currently recommended drugs for Chagas disease, sleeping sickness or visceral leishmaniasis on autophagy in the parasites, or the importance of autophagy in the manner by which the drugs exert their effects. However, it has recently been reported that a drug used for cardiac arrythmia in chronic Chagas patients administered in combination with a promising drug in Phase 2 of clinical trials also for Chagas treatment, induces autophagy and many other morphological defects in the cells. Although autophagy is probably upregulated as a rescue mechanism to protect the cell against the toxic effects of the drugs, the authors conclude that the parasites die as a result of autophagy [116].

Together, the data presented in this paper indicate that further research on autophagy in trypanosomatid parasites may provide important fundamental scientific data about a process that shows considerable differences with those of the well studied yeasts and mammals, and might be highly relevant for developing new chemotherapeutics against serious neglected tropical diseases.
